# Odonata of Canada

**DOI:** 10.3897/zookeys.819.25780

**Published:** 2019-01-24

**Authors:** Robert A. Cannings

**Affiliations:** 1 Royal British Columbia Museum, 675 Belleville St, Victoria, BC, V8W 9W2, Canada Royal British Columbia Museum Victoria Canada

**Keywords:** barcoding, biodiversity assessment, Biota of Canada, climate change, identification, Odonata, species at risk

## Abstract

Since Corbet’s thorough 1979 overview of Canadian Odonata, hundreds of regional works on taxonomy, faunistics, distribution, life history, ecology and behaviour have been written. Canada records 214 species of Odonata, an increase of 20 since the 1979 assessment. Estimates of unrecorded species are small; this reflects the well-known nature of the fauna. A major impetus for surveys and analyses of the status of species is the work of the Committee on the Status of Endangered Wildlife in Canada which provides a scientifically sound classification of wildlife species potentially at risk. As of 2017, six species have been designated “Endangered” and two “Special Concern” (only five of which are officially listed under the Federal Species at Risk Act (SARA)). The Order provides a good example of molecular barcoding effort in insects, as many well-accepted morphological species in Canada have been barcoded to some degree. However, more barcoding of accurately identified specimens of many species is still required, especially in most of the larger families, which have less than 70% of their species barcoded. Corbet noted that the larvae of 15 Canadian species were unknown, but almost all larvae are now well, or cursorily, described. Extensive surveys have greatly improved our understanding of species’ geographical distributions, habitat requirements and conservation status but more research is required to better define occurrence, abundance and biological details for almost all species.

Philip Corbet (1979), in his treatment of the Odonata in *Canada and its insect fauna*, noted that the order was well-known over much of the earth. Although this was true then, it is even more accurate today, for in the past four decades, dragonflies and damselflies have become the focus of much phylogenetic, behavioural, ecological, faunistic, and conservation study. Naturalists and photographers and others in the general public have taken a strong interest in the order and their documentation of distribution and status of species has greatly improved our knowledge of the group. Corbet also noted that the order was a small one and stated that “the world fauna probably does not greatly exceed the 5000 or so species now described”. Although today the Odonata is still considered a relatively small order of insects, it now consists of approximately 6000 named species in 30 families worldwide (Dijkstra et al. 2013). Estimates suggest that ca. 1000 to 1500 species remain to be named and, based on the fact that ca. 40 species have been described annually since 1970, 95% of the world’s species will probably be named by 2030 (Kalkman et al. 2008).

The order is predominantly tropical in distribution and is less diverse at higher latitudes. For example, as of October 2017, Canada recorded 214 species (Table 1; R Cannings unpubl. data) and the United States (including Alaska) listed 464, while Brazil had 736 species (DR Paulson pers. comm.). In addition, tropical countries, compared to temperate ones, generally have a much higher number of unrecorded and undescribed species.

**Table 1.** Census of Odonata in Canada.

Taxon^1^	No. species reported in Corbet (1979)	No. species currently known from Canada	No. BINs^2^ available for Canadian species	Est. no. undescribed or unrecorded species in Canada^3^	General distribution by ecozone^4^	Information sources^5^
**Order Odonata**						
**Suborder Zygoptera**						
Calopterygidae	4	5	4	0	Montane Cordillera, Taiga Plains, Boreal Shield, Hudson Plains, Newfoundland Boreal and ecozones southward, except Pacific Maritime	Walker 1953, 1958, Walker and Corbet 1975, Cannings et al. 1991, Brunelle 1997, Pilon and Lagacé 1998, Catling and Brownell 2000, Catling et al. 2004, 2005; AADIP, OC, NSC, specimens and databases from various collections
Lestidae	9	12	11	0	all ecozones except Arctic	see sources for Calopterygidae
Coenagrionidae	38	43	27	4	all ecozones including southern part of Arctic	see sources for Calopterygidae
**Suborder Anisoptera**						
Petaluridae	2^6^	1	0	1	Pacific Maritime, Western Interior Basin	Cannings 2002; NSC, OC, specimens in RBCM, UBC
Aeshnidae	24	24	25^7^	0	all ecozones including southern part of Arctic	see sources for Calopterygidae
Gomphidae	36	41	26	4	all ecozones except Taiga Cordillera and Arctic	see sources for Calopterygidae
Cordulegastridae	4	5	5	1	Pacific Maritime, Montane Cordillera, Western Interior Basin, Boreal Shield, Mixedwood Plains, Atlantic Maritime	see sources for Calopterygidae
Macromiidae	4	4	3	2	Pacific Maritime, Montane Cordillera, Western Interior Basin, Boreal Shield, Mixedwood Plains, Atlantic Maritime	see sources for Calopterygidae
Corduliidae	28	33	20	2	all ecozones including southern part of Arctic	see sources for Calopterygidae
Libellulidae	45	46	30	1	all ecozones including southern part of Arctic	see sources for Calopterygidae
**Total**	**194**	**214**	**151**	**15**		

^1^Classification follows that of Dijkstra et al. 2013; Catling et al. 2005. ^2^Barcode Index Number, as defined in Ratnasingham and Hebert (2013). ^3^Several unrecorded species may occur, especially in extreme southern central and eastern Canada; others might arrive from the USA in the next several years as their ranges expand. Undescribed species are unlikely. ^4^See Figure 1 in Langor (2019) for map of ecozones. ^5^ADIP – Atlantic Dragonfly Inventory Program, NSC – NatureServe Canada Conservation Data Centres (see http://www.natureserve.org/natureserve-network/canada/about-our-cdcs), OC – OdonataCentral (see http://www.odonatacentral.org), RBCM – Royal British Columbia Museum, UBC – University of British Columbia. See Cannings (2004) for a list of Canadian collections with significant holdings of specimens and data. ^6^*Tachopteryxthoreyi* (Hagen), originally reported from Québec, was discussed by Savard (1987) and discounted by Pilon and Lagacé (1998). ^7^Species and BINs do not completely align; see text for explanation.

Trueman and Rowe (2009) and Dijkstra et al. (2013) summarize the issues and relevant literature in Odonata phylogenetics; the latter outlines the most recent and probably the most widely accepted classification. Odonata is monophyletic and is divided into three suborders: Anisoptera (true dragonflies), Anisozygoptera and Zygoptera (damselflies), although some controversy over this classification remains (Dijkstra et al. 2013). Anisoptera and Zygoptera occur in Canada.

Canada has been a centre of odonatological research for at least 150 years. Léon Provancher (1874, 1878) studied the Québec fauna at least as early as the 1870s and Edmund Walker laboured for 70 years on important systematic and distribution works, especially monographs on *Aeshna* (1912) and *Somatochlora* (1925) and his monumental *The Odonata of Canada and Alaska* (Walker 1953, 1958) the last volume of which was completed by Philip Corbet (Walker and Corbet 1975). Walker’s important amateur collaborator, Francis Whitehouse (1941, 1948), primarily studied Odonata in Alberta and British Columbia. Corbet, along with Adrien Robert (1963) in Québec, acted as transitional researchers between the first half of the 1900s and the second half, when odonatology expanded dramatically in the nation.

The growth of organized international research and communication since the mid-1970s has stimulated study in Canada. This began with the creation of the International Odonatological Society (SIO) in Europe and its spread around the world. International symposia were held every two years; two were organized in Canada – Montréal (1979) and Calgary (1983). A Canadian newsletter, *Walkeria*, was disseminated twice a year from the mid-1980s to the late 1990s, when the society dissolved worldwide because of internal conflicts. Nevertheless, the SIO journals, *Odonatologica* and *Notulaeodonatologicae*, continue to be published. International activity in Canada largely switched in the 1990s to the Dragonfly Society of the Americas (begun in 1989) and the Worldwide Dragonfly Association (1997).

Cannings (2004) summarized the resources available to dragonfly and damselfly workers in Canada, including the most significant collections of specimens. On the North American scale, the most useful field books are Dennis Paulson’s (2009, 2011) guides to western and eastern species, which have eclipsed most others in quality and comprehensiveness; the distribution maps are small but carefully and accurately rendered. There are now dozens of other field guides available, some useful in both the USA and Canada; Lam (2004) is one of the best. Needham et al. (2000) and Westfall and May (2006) give the most detailed identification keys for adults and larvae of the continent’s fauna, and Garrison, von Ellenrieder and Louton (2006, 2010) provide illustrated keys and authoratative taxonomic summaries for all the New World genera. Corbet’s (1999) masterpiece, *Dragonflies: Behavior and Ecology of Odonata*, is the culmination of the long career of a prominent dragonfly biologist and is the critical resource for any research on the biology of the Odonata and its evolutionary context. The Internet is replete with valuable Odonata websites of all descriptions. OdonataCentral (http://www.odonatacentral.org/) disseminates information on distribution, biogeography, biodiversity, and identification of New World Odonata. Species distributions are mapped with submitted specimen and photograph records and an identification application based on the extensive database is available. The site hosts the web pages of the Dragonfly Society of the Americas, which publishes the journals *Argia* and the *Bulletin of American Odonatology* (with much Canadian content) and sponsors the official checklist of North American Odonata. The listserve Odonata-l (https://mailweb.ups.edu/mailman/listinfo/odonata-l) is a useful way to keep abreast of topics in the field.

Hundreds of regional works on taxonomy, faunistics, distribution, life history, ecology and behaviour have appeared since around the time of Corbet’s treatment. A few examples are mentioned here. In the West, Cannings and Stuart (1977) analyzed the British Columbia fauna and Cannings (2002) produced a British Columbia and Yukon guide for beginners. Cannings et al. (2000, 2007, 2008) undertook detailed inventories, from 1996 to 2005, jointly sponsored by the Royal British Columbia Museum and the British Columbia Conservation Data Centre. A provincial list and distribution maps for the province’s species are posted on E-Fauna BC (http://ibis.geog.ubc.ca/biodiversity/efauna/). The fauna of the largest provincial ecozone, the Montane Cordillera, was treated in Cannings and Cannings (2011); that of saline lakes in the province’s interior was investigated in Cannings and Cannings (1987) and the Odonata of a coastal glacial refugium was reported in Cannings and Cannings (1983). Many other publications document the British Columbia fauna; a few include Cannings et al. (1980), Paulson and Cannings (1980), and Simaika and Cannings (2004).

In the Prairie Provinces, two significant books have stimulated additional studies: Acorn (2004), a fine examination of the Zygoptera of Alberta; Hutchings and Halstead (2011), a field guide to the Odonata of the boreal forest of Saskatchewan. The most recent species lists for Alberta, Saskatchewan and Manitoba are those produced by the general status program (Canadian Endangered Species Conservation Council 2016), which are also available through the provincial Conservation Data Centres (NatureServe Canada: http://www.natureserve.org/natureserve-network/canada/about-our-cdcs). A Manitoba list (Hughes and Duncan 2003) gives additional data and the Manitoba Dragonfly Survey (http://www.naturenorth.com/dragonfly/) encourages the participation of naturalists in Odonata study. Numerous papers, including Acorn (1983), Hilton (1985), Hutchings (2004), Catling and Kostiuk (2004) and Hughes and Catling (2005) have helped improve our knowledge of the Odonata of the Great Plains. The predominant odonatologist of the prairies, Gordon Pritchard (e.g., 1989), authored, with his students, many elegant papers on the life histories and development of Odonata. Hornung and Rice (2003) studied wetland quality and Odonata in Alberta. The fauna of Canadian grasslands was summarized by Cannings (2014).

Ontario has been a leader in Odonata study ever since E.M. Walker’s superb work started the trend. Catling and Brownell (2000) published a summary of species and distribution that compliments the volumes of *Ontario Odonata* (Catling et al. 2000–2007), a discontinued annual summary of Odonata records published by the Toronto Entomologists’ Association. This publication also supplied notes on observations, range extensions and regional lists and is still a useful resource, PDF versions of which are available online (http://ontarioinsects.org/odonata_sum.htm). The Natural Heritage Information Centre, Ontario Ministry of Natural Resources and Forestry, maintains the provincial species list as well as the Ontario Odonata Atlas Database, which contains more than 80,000 records dating back to 1886. The field guides for southwestern Ontario (Carmichael et al. 2002) and Algonquin Provincial Park and environs (Jones et al. 2013) are examples of the detailed interest in Odonata study in Canada, as are websites such as those for Ojibway Prairie (Pratt 2013) and for regional Ontario lists (Pratt 2012). Many other publications document the Ontario fauna; a few include Skevington and Carmichael (1997), Cannings (1989, 2014), Catling (2001), and Jones and Burke (2004).

With the strong foundation of Provancher and Robert, Québec odonatology has flourished for many decades and, since the 1970s, *Fabreries* and *Nouv’Ailes* have been important sources of odonatological information; these are journals of L’Association des Entomologistes Amateurs du Québec. The major recent work on the province’s fauna is Pilon and Lagacé (1998). Entomofaune du Québec (http://entomofaune.qc.ca/entomofaune/odonates/odoindex.html) produces much valuable material on Québec odonates, including the provincial list and the atlas database. A preliminary atlas (Savard 2011) set the stage for a future, more comprehensive, biogeographical work. Hutchinson and Ménard (2014) is an excellent summary of Québec larvae. Numerous systematic notes and papers have appeared on the province’s fauna, from larval studies (e.g., Pilon and Legris 1987), general biology (Hutchinson 1991) and distributional works (Hutchinson and Ménard 1994) to phenology (Savard 1986), annotated lists (Ménard 1996, Perron et al. 2005) and reproduction (Hilton 1983, 1984).

The Atlantic Provinces have seen some of the most intensive Odonata surveying in Canada, thanks in large part to the contributions of amateurs in the past 20 years. However, Newfoundland and Labrador has a more boreal, less diverse fauna than the Maritime Provinces to the southwest and is not so well collected. Paul Brunelle amassed a dataset of records and inspired many of the region’s naturalists to collect data through the Atlantic Dragonfly Inventory Program (ADIP), which he launched in the early 1990s. That dataset contains ca. 37,500 records (including historical ones) representing 12,700 visits to 4,800 sites (PM Brunelle pers. comm.) and has vastly improved our understanding of species distribution and status in eastern Canada. Much of the data are available through the Atlantic Canada Conservation Data Centre and, in the near future, will be housed, along with the specimens, at the New Brunswick Museum. Brunelle (1997) set the stage for this odonatological renaissance, and his superb treatment of species diversity in the Atlantic Maritime Ecozone (Brunelle 2010) is a testament to the success of ADIP. Many other publications on the region have appeared, of course, including those on distribution (Hilton 1990), population dynamics (Conrad and Herman 1996), habitat/ecology (Catling et al. 2006) and important new records (e.g., Harding 2007). A useful website on the fauna of New Brunswick is at http://www.odonatanb.com/.

The fauna of the territories has not escaped notice. Cannings et al. (1991) and Cannings and Cannings (1997) documented the surveys organized by the Biological Survey of Canada in the Yukon. Catling (2003) produced an atlas of the species in the Northwest Territories and, subsequently, an annotated checklist (Catling et al. 2004). In Nunavut, with its poor access and low Odonata diversity, there has been hardly any collecting. Only a few specimens of six species have been recorded north of treeline, including the James Bay islands. There have been no surveys in the potentially productive boreal forest in the southwest corner of the Territory.

Treatments of Odonata in particular habitats include those of Canadian peatlands and marshes (Hilton 1987), peatlands of the northern Cordillera (Cannings and Cannings 1994), saline lakes in British Columbia (Cannings and Cannings 1987) and Canadian grasslands (Cannings 2014).

The first comprehensive published annotated list of Canadian Odonata, Catling et al. (2005), listed 208 species and included the first general status ranks produced by the National General Status Program of Environment and Climate Change Canada. At the end of 2017, the number of species recorded in Canada (214) has increased by 20 since Corbet’s 1979 assessment (Table 1). All families show increases except the Aeshnidae (unchanged) and the Petaluridae (decrease from 2 to 1 owing to an error in the interpretation of specimen data). Seven species are considered vagrants or wanderers and presumably do not breed in Canada, although they may appear year after year. A few of the additions to the national list are rare, hard-to-find species that probably have been in Canada a long time, e.g., *Somatochlorahineana* Williamson, *Williamsonialintneri* (Hagen); more have recently moved northwards from the USA (e.g., *Archilestescalifornicus* McLachlan). Estimates of unrecorded species are small (Table 1) and reflect the well-known nature of the fauna. Several species, especially in extreme southern central and eastern Canada, probably occur but have not yet been recorded or might arrive from the USA in the next few years. An example is *Enallagmadivagans* Selys, which occurs just across the USA border near Detroit (CD Jones pers. comm.). One species, the gomphid *Stylurusplagiatus* Selys, formerly known from Ontario, has apparently been extirpated from the country (Canadian Endangered Species Conservation Council 2016). *Crocothemisservilia* (Drury), an Asian libellulid established in Florida and the only odonate introduced to the New World, was imported to Québec in a shipment of aquatic plants kept indoors (Perron et al. 2003). The record was rejected by Catling et al. (2005) and, although *C.servilia* is listed in Wild Species 2015 (Canadian Endangered Species Conservation Council 2016) as the first and only alien species in Canada, it is not accepted herein. The sole species discovered in Canada and first described from Canadian material since the 1979 assessment is *Neurocorduliamichaeli* Brunelle (Brunelle 2000), a crepuscular corduliid from eastern Canada.

The Order Odonata provides a good example of DNA barcoding effort in a group of insects. Many well-accepted Canadian species (based on morphological and reproductive characters) have been DNA barcoded to some degree. Barcode Index Numbers (BINs) are clusters of barcode sequences that usually show concordance with species; the system therefore can be used to verify species identifications (Ratnasingham and Hebert 2013). Table 1 suggests that BINs are available for approximately 70% of the Odonata species recorded in Canada. Most species that are sequenced correspond reasonably well with BINs. However, some anomalies are hidden in the numbers. For example, in the Aeshnidae, which appears to have all 24 species represented by BINs, at least four species are not included and some BINs are not linked to species. Some BINs suffer from containing too few sequences. Results show that the Odonata is susceptible to BINs not aligning with recognized species, either by lumping well-known morphological species in a single BIN or by dividing a single species into several separate BINs. Although I know of no studies analyzing DNA barcoding in Odonata, overviews in other taxa, such as bees (Sheffield et al. 2017), show similar results. In Canadian Odonata, distinct morphological species, *Aeshnainterrupta* and *A.eremita*, are placed in a single BIN. Both are common, transcontinental species and whereas the latter is morphologically similar across its wide range, the former has three subspecies, although these are problematic (Catling et al. 2005). *Aeshnaumbrosa*, another common, transcontinental species, is morphologically uniform over most of North America; however, west of the Rockies, it has an additional colour form, which has been considered a subspecies. Material from across Canada is assigned to a single BIN, except in New Brunswick, where sequences are divided into several separate BINs, even though current taxonomic understanding suggests cryptic species would be highly unlikely. Clearly, additional work is required to resolve these questions. The numbers presented in Table 1 for some other families likely represent anomalies similar to those found in the Aeshnidae. More barcoding of more well-identified specimens of many species is still required, especially in most of the larger families, which indicate only moderate completion: Coenagrionidae (63%), Gomphidae (63%), Corduliidae (61%), and Libellulidae (65%). Currently available molecular data do not suggest the possibility of undescribed cryptic species. Despite some problems, the Odonata, unlike some other orders, is known well enough that, with some concentrated work, all Canadian species might ultimately be supported with DNA barcodes.

A major impetus for surveys and analyses of the status of Odonata species is the work of the Committee on the Status of Endangered Wildlife in Canada (COSEWIC) (http://www.cosewic.gc.ca/default.asp?lang=en&n=F3AE41D5-1#) which provides a scientifically sound classification of wildlife species potentially at risk. Under the Species at Risk Act (SARA), COSEWIC serves as an independent body of experts responsible for identifying and assessing such species. COSEWIC produces comprehensive status reports of species and results are reported to the Canadian government and the public; if the Minister of Environment and Climate Change designates the species under Schedule 1 of the Act, the species may then qualify for legal protection and recovery under SARA. Assessments of Odonata began in 2004. As of 2018, five species have been designated as Endangered: *Phanogomphusquadricolor* (Walsh) (Ontario), *Gomphurusventricosus* (Walsh) (New Brunswick), *Somatochlorahineana* Williamson (Ontario), *Stylurusamnicola* (Walsh) (Ontario) and *Stylurusolivaceus* (Selys) (British Columbia). *Ophiogomphushowei* Bromley (Ontario, New Brunswick) is designated Special Concern. Two species have COSEWIC status but have yet to be designated under SARA: *Styluruslaurae* Williamson (Ontario: Endangered) and *Argiavivida* Hagen (British Columbia and Alberta: Special Concern) (Species at Risk Public Registry: http://www.registrelep-sararegistry.gc.ca/sar/index/default_e.cfm). An associated national effort is the General Status of Species in Canada. A report, *Wild Species*, is produced every five years. Odonata were first included in 2005; the most recent report was produced in 2015 (Canadian Endangered Species Conservation Council 2016). Occurrence and status for each odonate species known in Canada is given for all provinces and territories. The list is slightly out-of-date; 213 species are documented.

The taxonomy of the Nearctic Odonata is relatively well-known compared to that of many other insect groups. In Canada, certain closely related pairs of taxa such as *Erythemiscollocata* (West) and *E.simplicicollis* (East) and, especially, *Amphiagrionabbreviatum* (West) and *A.saucium* (East) require more study to ascertain if they should remain separate species. Phylogenetic examination lumped the widespread *Sympetrumoccidentale* Bartenev (West) with *S.semicinctum* (East) (Pilgrim and von Dohlen 2007) but further work on these and other such variable species in Canada is desirable. *Aeshnainterrupta* is another good example of a species with widespread geographic variation (Catling et al. 2005). Genetic work may also help determine the relationships among Palaearctic and Nearctic species, as was done with the separation of the Nearctic *Enallagmaannexum* from the Palaeartic *E.cyathigerum* (Charpentier) (Turgeon et al. 2005).

The identification of adults of both sexes has been significantly enhanced by the many excellent field guides and photo websites produced by experienced field biologists (see above). Although not specific to odonates, BugGuide (https://bugguide.net) and iNaturalist (inaturalist.ca) offer photograph identification services and help improve distributional knowledge. Cellphone applications are popular; Birdseye (http://www.birdseyebirding.com/apps/dragonfly-id-app/) produces a comprehensive one based on data from OdonataCentral, the premier site for Odonata distribution in North America.

Corbet (1979) noted that the larvae of 15 Canadian species were unknown, but most of these are now documented in detail (e.g., Cannings and Doerksen 1979, Charlton and Cannings 1993, Kenner et al. 2000). Thus, almost all larvae are now well, or cursorily, described (K Tennessen pers. comm.) Larvae of all Canadian Zygoptera have been described or characterized; in the latter category are a few diagnosed only in the keys in Westfall and May (2006). Larval descriptions and identification keys can be improved; those dealing with instars younger than the final one are particularly needed.

Most gaps in knowledge indicated by Corbet in the 1979 synopsis still need work. Our understanding of most species’ geographical distribution, habitat requirements and conservation status has been greatly improved since 1979 owing to the extensive surveys and amateur observations noted above. However, more research is required to better define occurrence, abundance and biological details for almost all species of odonates. Detailed, annotated site lists developed over several years would be extremely valuable in all regions, as would autecological research on species to determine habitat requirements. In the face of climate change, baseline data on distribution and habitat (with detailed vegetation and water characteristics) are of high value. For example, Cannings et al. (2016) discuss the range expansion of *Libellulapulchella* (Drury) in British Columbia, Alberta and Saskatchewan in the context of the proliferation of man-made ponds and other wetlands as well as climate warming. They note that, in addition, recent wet conditions have created more suitable habitat for this dragonfly on the western Great Plains. The continuous monitoring of selected study sites for changes in species composition and habitat details would be most useful. Monitoring of conservation status is a priority as habitats and climate fluctuate in character. Studies examining the effects of disturbance and habitat change on species are needed. As indicated above, COSEWIC has studied several species and more status reports from this national committee, or allied provincial agencies, will be required if drying wetlands and reduced stream flows begin to affect populations of rare species.
